# Involvement of multiple myeloma cell-derived exosomes in osteoclast differentiation

**DOI:** 10.18632/oncotarget.3830

**Published:** 2015-04-12

**Authors:** Lavinia Raimondi, Angela De Luca, Nicola Amodio, Mauro Manno, Samuele Raccosta, Simona Taverna, Daniele Bellavia, Flores Naselli, Simona Fontana, Odessa Schillaci, Roberto Giardino, Milena Fini, Pierfrancesco Tassone, Alessandra Santoro, Giacomo De Leo, Gianluca Giavaresi, Riccardo Alessandro

**Affiliations:** ^1^ Laboratory of Tissue Engineering - Innovative Technology Platforms for Tissue Engineering (PON01-00829), Rizzoli Orthopedic Institute, Palermo, Italy; ^2^ Department of Experimental and Clinical Medicine, Magna Graecia University and Medical Oncology Unit, T. Campanella Cancer Center, Salvatore Venuta University Campus, Catanzaro, Italy; ^3^ Institute of Biophysics, National Research Council of Italy, Palermo, Italy; ^4^ Section of Biology and Genetics, Department of Biopathology and Medical Biotechnology, University of Palermo, Italy; ^5^ Rizzoli Orthopedic Institute, Bologna, Italy; ^6^ Laboratory of Preclinical and Surgical Studies, Rizzoli Orthopedic Institute, Bologna, Italy; ^7^ Divisione di Ematologia A.O. Ospedali Riuniti Villa Sofia-Cervello, Palermo; ^8^ Institute of Biomedicine and Molecular Immunology (IBIM), National Research Council of Italy, Palermo, Italy

**Keywords:** exosomes, multiple myeloma, osteoclasts, tumor microenvironment

## Abstract

Bone disease is the most frequent complication in multiple myeloma (MM) resulting in osteolytic lesions, bone pain, hypercalcemia and renal failure. In MM bone disease the perfect balance between bone-resorbing osteoclasts (OCs) and bone-forming osteoblasts (OBs) activity is lost in favour of OCs, thus resulting in skeletal disorders. Since exosomes have been described for their functional role in cancer progression, we here investigate whether MM cell-derived exosomes may be involved in OCs differentiation. We show that MM cells produce exosomes which are actively internalized by Raw264.7 cell line, a cellular model of osteoclast formation. MM cell-derived exosomes positively modulate pre-osteoclast migration, through the increasing of CXCR4 expression and trigger a survival pathway. MM cell-derived exosomes play a significant pro-differentiative role in murine Raw264.7 cells and human primary osteoclasts, inducing the expression of osteoclast markers such as Cathepsin K (CTSK), Matrix Metalloproteinases 9 (MMP9) and Tartrate-resistant Acid Phosphatase (TRAP). Pre-osteoclast treated with MM cell-derived exosomes differentiate in multinuclear OCs able to excavate authentic resorption lacunae. Similar results were obtained with exosomes derived from MM patient's sera. Our data indicate that MM-exosomes modulate OCs function and differentiation. Further studies are needed to identify the OCs activating factors transported by MM cell-derived exosomes.

## INTRODUCTION

More than 70% of patients affected by multiple myeloma (MM) develop severe osteolytic bone disease, which negatively affects the quality of life due to the occurring pathological fractures, spinal cord compression, pain and hypercalcemia [1, 2]. Even though, rapid advances in the therapeutic options in bone disease treatment have been accomplished in the recent years, the progression of MM bone disease is rarely halted. Additional studies to understand the complex mechanisms adopted by MM cells to influence tumor microenvironment will be needed to overcome the limits of current pharmacology [3-6].

MM bone disease is sustained by a supportive microenvironment based on complex cross-talk involving stromal, immune, endothelial and bone cells, as well as extracellular matrix components. In bone marrow, MM cells modify the normal microenvironmental conditions and in turn require host modifications for their survival [7]. In contrast to normal bone remodelling, during multiple myeloma progression the functional balance between osteoclasts (OCs) and osteoblasts (OBs) is definitively perturbed. Differently by other metastatic diseases such as breast and prostate cancer, where both OCs and OBs activity is increased, in MM increased osteoclastic activity is the major element of bone disease [2, 8]. The increased number and activity of OCs further promote MM progression by both direct and indirect mechanisms, thus maintaining a vicious cycle between bone destruction and tumor cell survival [9]. MM cells directly stimulate OCs differentiation and activation by secreting local osteoclast activating factors (OAFs) and indirectly by stimulating the cells of tumor microenvironment, such as bone marrow stromal cells (BMSCs) and OBs, to secrete OAFs [9]. Among the main cytokines involved in this crosstalk are: interleukin-6, interleukin-1β, interleukin-3, TNF-α, TGF-1β, activin A, and dickkopf (DKK)-1 [10-12]. The receptor activator of NF-κB (RANK), its ligand RANKL, and the decoy receptor for RANKL, osteoprotegerin (OPG), play a crucial role in bone remodelling, inducing differentiation and survival of osteoclasts [13]. In MM, deregulation of RANKL to OPG ratio leads to typical osteolysis of bone disease; however, the direct RANKL expression by human myeloma cells is controversial and several reports suggested that human MM cells do not directly produce RANKL but rather induce its expression in stromal cells [14-16].

In the last years, new actors of tumor microenvironment crosstalk have been identified.

Exosomes are small membrane vesicles (40-150 nm) of endocytic origin secreted by most cells and thoroughly described for their functional relevance in cancer, being now recognized as a novel mode of cell-cell communication during tumor growth and progression. A number of studies have showed that exosomes released by cancer cells may affect survival, apoptosis, invasion, angiogenesis and resistance to chemotherapy as well as prepare the metastatic niche [17].

These nanovesicles actively transport and transfer information such as proteins, microRNAs and mRNAs to target cells, thus influencing their behaviour and strongly modifying the entire microenvironment [18]. Several reports showed that the serum of patients affected by cancer is characterized by high levels of exosomes and their quantity seems to correlate with the malignant behaviour of cancer [19-21]. In MM, cell-derived microvesicles are considered mediators for myeloma angiogenesis, while BMSC-derived exosomes significantly act on viability, survival, migration and drug resistance of MM cells [22, 23]. On these premises, we hypothesized that MM cell-derived exosomes might play a relevant functional role in OCs differentiation. MM cell-derived exosomes were firstly isolated and characterized *in vitro* and then their biological effects were evaluated in murine macrophage Raw264.7 cells and human primary osteoclasts. Our results clearly show that multiple myeloma cells release exosomes that in turn support both viability and migration of osteoclast precursors (pOCs) as well as their function and differentiation in giant and multinucleated osteoclasts. Similar results were obtained with exosomes derived from MM patient's sera. In summary, a more detailed understanding about the molecular mechanisms underlying exosomes-mediated bone disease may open new opportunities for combinatory therapeutical approaches as well as could lead to the identification of bone disease-biomarkers in MM.

## RESULTS

### MM-derived exosomes characterization and internalization in Raw264.7 cells

**Figure 1:**
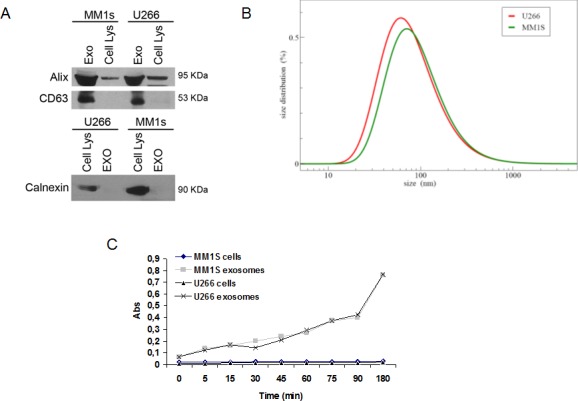
Characterization of exosomes released by multiple myeloma cells **A.** Western blotting analysis of Alix, CD63 and Calnexin in both U266, MM1s-derived exosomes and cellular lysates. **B.** Dynamic light scattering (DLS) analysis of U266 and MM1s-derived exosomes **C.** Acetylcholinesterase assay of exosomes and cell lysates obtained from U266 and MM1s cells.

Exosomes produced by three MM cell lines (U266, MM1S and OPM2) were characterized by western blot analysis. Figure 1A (upper panel) shows that U266- and MM1s-cell derived exosomes abundantly expressed Alix and CD63, while Calnexin, an ubiquitously expressed ER protein, was exclusively found in cellular fractions (Figure 1A, lower panel). Similar results were obtained with OPM2-derived exosomes (Suppl. Figure 1A). The DLS analysis showed an average hydrodynamic diameter of about 100 nm for U266- and MM1s-cell-derived exosomes and 50 nm for OPM2-derived exosomes (Figure 1B; Suppl. Figure 1B). We then tested the activity of acetylcholinesterase, an enzyme known to be enriched in exosomes, and we observed an increased activity in the extracellular nanovesicles (Figure 1C; Suppl. Figure 1C) [24].

**Figure 2:**
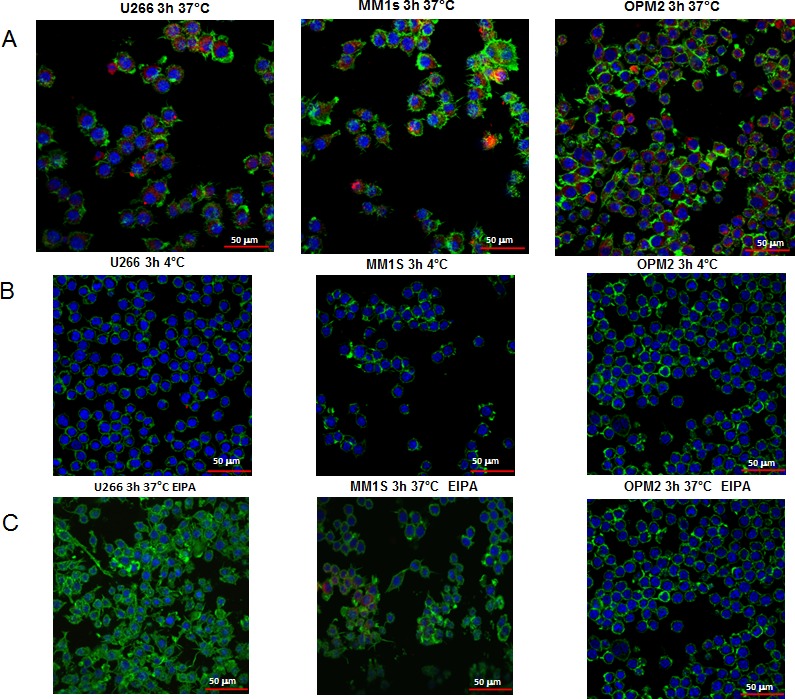
Uptake of multiple myeloma cell-derived exosomes by osteoclasts precursors **A.** Analysis at confocal microscopy of Raw264.7 cells treated for 3 hours with 25 μg/ml of U266, MM1s and OPM2 exosomes. Raw264.7 cells were stained with Actin green (green), nuclear counterstaining was performed using Hoescht (blue) and exosomes were labelled with PKH26 (red). **B.** To evaluate whether exosomes uptake was a biologically active process, Raw264.7 cells treated with 25 μg/ml of U266, MM1s and OPM2 exosomes were incubated at 4°C **C.** To evaluate whether exosomes uptake was mediated by endocytosis in an energy-dependent process, Raw264.7 cells were treated for 3 hour with 25 μg/ml of exosomes and EIPA (25 μM), Scale bar = 50 μm.

MM cell-derived exosomes labeled with PKH-26 were internalized by the murine macrophage cell line Raw264.7 after incubation of 3 hours at 37°C. Figure 2A shows a typical perinuclear localization of internalized exosomes. The up-take of exosomes in Raw264.7 cells was inhibited by incubation at 4°C (Figure 2B), as well as by EIPA treatment (Figure 2C). Semi-quantitative analysis of PKH-26 fluorescence intensity in the cytoplasm of Raw264.7 cells confirmed the imaging data (Suppl. Figure 2).

### MM cell-derived exosomes support migration of pOCs cells

Since, in bone disease, myeloma cells exert relevant effects on recruitment and proliferation of OC progenitors, here we investigated if MM cell-derived exosomes may modulate the proliferative and migratory properties of Raw264.7 cells. Cell viability analysis showed that U266- and MM1s-derived exosomes induced only a slight increase in Raw264.7 cell proliferation within 72 hours (Suppl. Figure 3A, upper panel) and a decrease after 6 days of exposure when induction of mature osteoclasts differentiation occurred (Suppl. Figure 3A, lower panel). OPM2-derived exosomes did not affect Raw264.7 cell viability (Suppl. Figure 3B).

**Figure 3:**
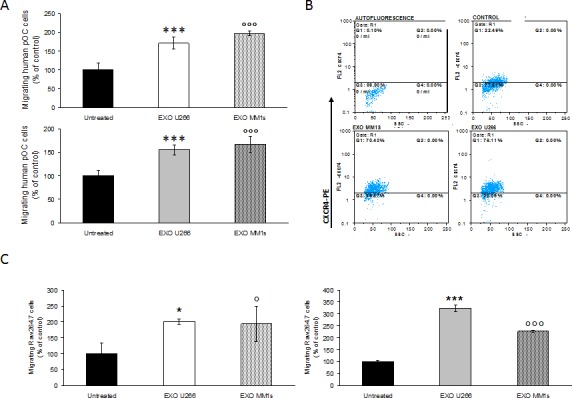
Multiple myeloma cell-derived exosomes induce migration of osteoclasts precursors **A.** Migration assay of human pOCs untreated or pretreated for 24 hours with 25 μg/ml of U266 and MM1s exosomes (upper panel); in the lower panel is shown the motility of human pOCs stimulated by addition of 25 μg/ml of U266 and MM1s exosomes in the bottom chamber of the transwell (lower panel). Sidak test: ^*^,EXO-U266 *vs* Untreated (^*^p < 0.01); °,EXO-MM1s *vs* Untreated (°*p* < 0.05). **B.** Flow cytometry analysis of CXCR4 in human pOCs untreated (control) or pretreated for 24 hours with 25 μg/ml of U266 and MM1s exosomes **C.** Migration assay of Raw264.7 cells untreated or pretreated for 24 hours with 25 μg/ml of U266 and MM1s exosomes (left panel). In the right panel is shown the motility of Raw264.7 cells stimulated by addition of 25 μg/ml of U266 and MM1s exosomes in the bottom chamber of the transwell. Sidak test: ^*^,EXO-U266 *vs* Untreated (^*^p < 0.01); °,EXO-MM1s *vs* Untreated (°*p* < 0.05).

The role of MM cell-derived exosomes on osteoclast precursors (pOCs) migration was investigated by a transwell chamber chemotaxis assay. Notably, we found that a 24h pretreatment of human pOCs with U266 and MM1s cell-derived exosomes increased their migratory attitudes (Figure 3A, upper panel), presumably via an increase of CXCR4 expression (Figure 3B).

Similarly, the number of Raw264.7 cells migrated across the 8-μm pore-size membrane increased when cells were pretreated for 24h with U266- or MM1s cell-derived exosomes (Figure 3C, left panel).

Finally, both human pOCs (Figure 3A, lower panel) and Raw264.7 cells (Figure 3C, right panel) were induced to migrate by MM exosomes, when MM vesicles were added to the bottom of the transwells. OPM2 cell-derived exosomes affected Raw264.7 migration less efficiently (Suppl. Figure 3C, upper and lower panel). These findings indicate that exosomes released by myeloma cells can influence both murine and human pOCs migration.

### Effect of multiple myeloma cell-derived exosomes on osteoclast gene expression

**Figure 4:**
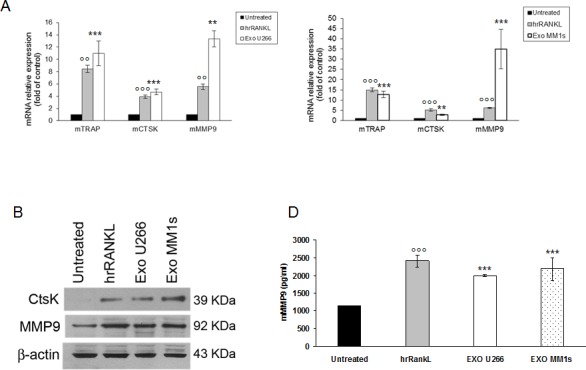
Multiple myeloma cell-derived exosomes increase expression of osteoclasts specific markers **A.** Quantitative RT-PCR of mTRAP, mCTSK and mMMP9 in Raw264.7 cells untreated or treated for six days with 25ng\ml of hrRANKL (positive control) or with 25 μg/ml of U266 (left panel) and MM1s exosomes (right panel). Values are expressed as fold of control and are the mean of three different experiments. Sidak test: ^*^,EXO-U266/MM1s *vs* Untreated (^*^p < 0.05); °,hrRANKL *vs* Untreated (°*p* < 0.05); ^+^ **B.** Western blotting analysis of CTSK and MMP9 in Raw264.7 cells untreated or treated for six days with 25ng\ml of hrRANKL (positive control) or with 25 μg/ml of U266 and MM1s exosomes. β-actin was used as loading control. **C.** Murine Metalloproteinase-9 (mMMP9) protein levels in the conditioned medium derived from Raw264.7 cells untreated or treated for six days with 25ng\ml of hrRANKL (positive control) or with 25 μg/ml of U266 and MM1s exosomes were determined by ELISA. Data presented are the mean of three different experiments. Sidak test: ^*^,EXO-U266/MM1s *vs* Untreated (^*^p < 0.05); °,hrRANKL *vs* Untreated (°*p* < 0.05).

qRT-PCR analysis revealed that stimulation of murine macrophages with RANKL increased, as already known, the expression of OCs specific markers; notably, U266 and MM1s exosomes markedly induced expression of TRAP and CTSK as compared to control cells. In particular, MMP9 mRNA expression was significantly higher in Raw264.7 cells treated with exosomes as compared to untreated cells or RANKL-treated cells (Figure 4A). Consistently, we observed a dose dependent effect of MM cell-derived exosomes on the mRNA levels of OCs markers as well as a reduced effect of the corresponding conditioned medium deprived of exosomes (Suppl. Figure 4A-4B).

The results were confirmed also by western blotting analysis for CTSK and MMP9 (Figure 4B). The increase of expression was statistically significant but less evident in cells treated with OPM2 exosomes (Suppl. Figure 3D). Taking in account that MMP9 protein secretion is increased in differentiated OCs, we investigated whether MM cell-derived exosomes were able to increase murine MMP9 secretion in pre-OCs cells. Figure 4C shows that Raw264.7 cells treated with U266 and MM1s exosomes significantly released mMMP9 protein in culture medium. All together these findings indicate that MM cell-derived exosomes directly induce the expression of specific OCs markers and modulate the secretion of proteases involved in bone resorption activity.

### Multiple myeloma cell-derived exosomes induce osteoclast formation and stimulate bone resorption activity in mature osteoclast-like cells

**Figure 5:**
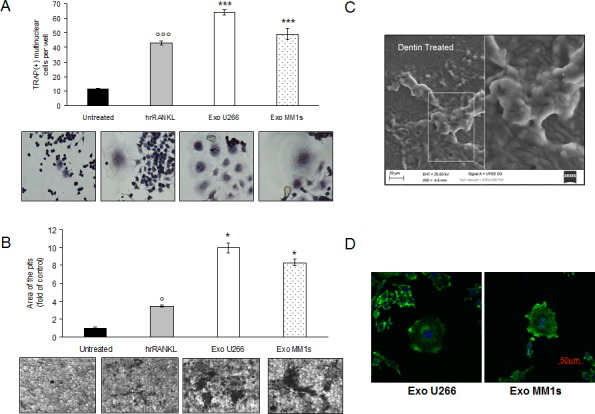
Exosomes produced by multiple myeloma cells induce osteoclast formation in RAW 264.7 cells and promote formation of lacunae on dentine slices **A.** Raw264.7 cells incubated with 25ng\ml of hrRANKL (positive control) or with 25 μg/ml of U266 and MM1s exosomes for six days, and then stained for TRAP expression. TRAP positive cells were photographed and counted. Lower panel shows representative pictures of TRAP^+^Raw264.7 cells (original magnification 20x). Data presented are the mean of three different experiments. Sidak test: ^*^,EXO-U266/MM1s *vs* Untreated (^*^p < 0.05); °,hrRANKL *vs* Untreated (°*p* < 0.05); **B.** Bone resorption ability of Raw264.7 cells treated for six days with 25ng\ml of hrRANKL (positive control) or with 25 μg/ml of U266 and MM1s exosomes was evaluated by resorption pit assay on dentine discs. Data relative to the resorbed areas by mature osteoclasts are represented as fold increase of Raw264.7 cells untreated (black column, 1 arbitrary unit). Data presented are the mean of three separate experiments. Sidak test: *,EXO-U266/MM1s *vs* Untreated (*p < 0.05); °,hrRANKL *vs* Untreated (°p < 0.05); Lower panel shows representative pictures of lacunae (dark areas) formed by cells treated with hrRANKL or MM-exosomes (original magnification 20x). **C.** Representative scanning electron microscopy (SEM) image of resorption pits generated by Raw264.7 cells cultured on dentine slices and treated with MM exosomes. Scale bar = 20 μm. **D.** Confocal microscopy of Raw264.7 cells cultured for six days with 25 μg/ml of U266- or MM1s-derived exosomes and fixed; actin filaments in podosomes were labeled using Alexa Fluor^®^ 488 phalloidin (green) and nuclei were counterstained with DAPI (blue). Scale bar = 50 μm.

We evaluated if exosomes released by myeloma cells might control OCs formation and OCs activity. TRAP staining and the formation of multinucleated cells were used as markers of differentiated cells. As showed in Figure 5A, treatment of Raw264.7 cells with exogenous RANKL confirmed the presence of TRAP-positive multinucleated OCs (Figure 5A, lower panel). Importantly, in the presence of MM cell-derived exosomes, the number of TRAP-positive multinucleated OCs was significantly higher compared to control cells (Figure 5A, upper panel). The strongest effect was observed with U266-derived exosomes, reaching a ten fold increase in the size of multinucleated OCs compared to control cells. We next investigated the ability of Raw 264.7 cell-derived OCs to resorb bone by stimulating for 6 days OCs formation on synthetic dentine discs with RANKL or MM cell-derived exosomes (Figure 5B, upper and lower panel). After removal of adherent cells, dentine discs were examined for the presence of resorption lacunae by dark field microscopy. OCs induced by MM cell-derived exosomes had a marked ability to resorb dentin substrate (dark areas); in particular, stimulation with exosomes increased not only the number of pits but also the digestion areas, which resulted larger compared to control cells (Figure 5B, lower panel). In Figure 5C is shown a scanning electron microscopy (SEM) image of a typical bone resorption area obtained by OCs cultured on synthetic dentine disc and treated with MM cell-derived exosomes. To further confirm the exosome induced phenotype of the cells, we also observed osteoclast morphology by staining cells with Alexa fluor 488-phalloidin that detects actin filaments in podosomes. As evident in Figure 5D, osteoclasts induced by MM cell-derived exosomes exhibited features of functional resorptive cells such as actin rings. To finally confirm that the effect of MM-cell derived exosomes on osteoclastogenesis was cell-type specific, we treated Raw264.7 cells with exosomes derived by SW620 cells, a highly aggressive metastatic colorectal cancer cell line which usually not metastasize to bone. Exosomes produced by SW620 cells were characterized by western blot analysis (Suppl. Figure 5A) and DLS analysis (Suppl. Figure 5B), thus confirming exosome identity.

**Figure 6:**
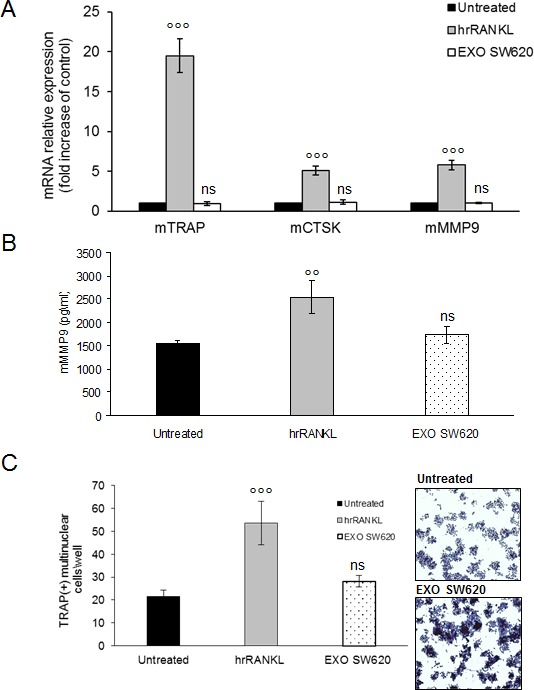
Colorectal cancer cell-derived exosomes not affect osteoclastogenesis of RAW 264.7 precursors **A.** Quantitative RT-PCR of mTRAP, mCSTK and mMMP9 in Raw264.7 cells untreated or treated for six days with 25ng\ml of hrRANKL (positive control) or with 25 μg/ml of SW620 exosomes. Values are expressed as fold of control and are the mean of three different experiments. Sidak test: ^*^,EXO-SW620 *vs* Untreated (^*^p < 0.05); °,hrRANKL *vs* Untreated (*p* < 0.05); **B.** Murine Metalloproteinase-9 (mMMP9) protein levels in the conditioned medium derived from Raw264.7 cells untreated or treated for six days with 25ng/ml of hrRANKL (positive control) or with 25 μg/ml of SW620 exosomes were determined by enzyme-linked immunoadsorbent assay (ELISA). Data presented are the mean of three separate experiments. Sidak test: ^*^,EXO-SW620 *vs* Untreated (*p* < 0.05); °,hrRANKL *vs* Untreated (°*p* < 0.05); statistically not significative: ns. **C.** RAW 264.7 cells incubated with 25ng/ml of hrRANKL (positive control) or with 25 μg/ml of SW620 for six days, and then stained for TRAP expression. TRAP positive cells (i.e., those containing three nuclei) were photographed and counted. Lower panel shows representative pictures of TRAP^+^Raw264.7 cells (original magnification 10x). Data presented are the mean of three separate experiments. Sidak test: ^*^,EXO-SW620 *vs* Untreated (^*^p < 0.05); °,hrRANKL *vs* Untreated (°*p* < 0.05); statistically not significative: ns.

The results showed in Figure 6A clearly indicate that the expression of OCs specific markers did not change after SW620 cell-derived exosomes treatment; also mMMP9 secreted by OCs cultured with SW620 cell-derived exosomes was not significative (Figure 6B). Furthermore, TRAP-staining assay revealed that SW620-exosomes did not increase OCs formation (Figure 6C). In summary, these data demonstrate that MM cell-derived exosomes specifically mediate the differentiation of pre-OCs, promoting their bone resorptive activity.

### Multiple myeloma cell-derived exosomes inhibit apoptosis of pOCs cells and promote their survival

**Figure 7:**
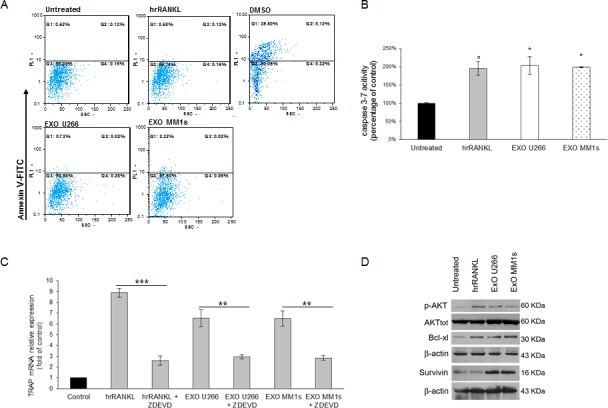
Exosomes released by multiple myeloma cells support osteoclast precursors survival and inhibit apoptosis **A.** Facs analysis of Annexin V iodide staining of Raw264.7 cells treated for 24 hours with 25ng\ml hrRANKL, 1% DMSO or 25 μg/ml of U266- and MM1s-derived exosomes **B.** Caspase 3/7 activity assay was performed in Raw264.7 cells untreated or treated for 24 hours with 25ng\ml of hrRANKL and with 25 μg/ml of U266- and MM1s-derived exosomes. Luminescence was measured using the Caspase 3/7 Glo assay. Results are expressed as percentage of caspase activity compared to untreated cells. Averaged values of three independent experiments are plotted including ±S.D. Sidak test: ^*^,EXO-U266/MM1s *vs* Untreated (^*^p < 0.05); °,hrRANKL *vs* Untreated (°p < 0.05); **C.** Quantitative RT-PCR of mTRAP in Raw264.7 cells untreated or pretreated with a caspase-3 inhibitor (50μM Z-DEVD-FMK) 1h before and during hrRANKL or MM-exosomes treatment. Values are expressed as fold of control and are the mean of three different experiments. Sidak test: ^*^,EXO-U266/MM1s + ZDEVD *vs* EXO-U266/MM1s (^*^p < 0.05); °,hrRANKL+ZDEVD *vs* hrRANKL (°p < 0.05); ^+^ **D.** Western blotting analysis of p-AKT, TOT-AKT, Bcl-xl and Survivin in Raw264.7 cells untreated or treated with 25ng\ml of hrRANKL (positive control) or with 25 μg/ml of U266 or MM1s exosomes (Exo U266/MM1s). β-actin was used as loading control.

We next examined, by Annexin V staining, whether apoptotic events occurred in Raw264.7 cells cultured with MM cell-derived exosomes. As shown in Figure 7A, MM cell-derived exosomes, as well as hrRANKL, did not induce apoptosis in Raw264.7 cells, differently by treatment with DMSO, a known apoptogen and activator of caspase-3 activity. Notably, we found that treatment of cells with MM cell-derived exosomes was associated with caspase 3 activation as revealed by increased caspase 3/7 activity (Figure 7B).

As Szymczyk KH and colleagues [25] reported that induction of differentiation in Raw264.7 cells is dependent on caspase-3 activity, we next investigated if OCs differentiation induced by MM cell-derived exosomes may require caspase-3 activation. We pharmacologically inhibited caspase-3 activity in Raw264.7 cells exposed to MM cell-derived exosomes (Suppl. Figure 6A) and then we analyzed TRAP activity; interestingly, we observed that inhibition of caspase-3 activity reduced TRAP mRNA expression in Raw264.7 cells treated with MM cell-derived exosomes (Figure 7C) as well as the number of TRAP positive cells after 4 days of treatment (Suppl. Figure 6B).

Western blotting analysis revealed an increase of anti-apoptotic protein BCl-Xl levels, survivin levels and phospho-AKT levels (Figure 7D). Our data were confirmed by the lack of TUNEL staining and nuclear condensation in Raw 264.7 cells treated with exosomes (data not shown). Taken together, these findings provide evidence that MM cell-derived exosomes exert pro-differentiative effects on pOCs cells by inhibiting apoptotic mechanisms.

### Exosomes isolated from plasma of patients with multiple myeloma modulate osteoclasts differentiation

**Figure 8:**
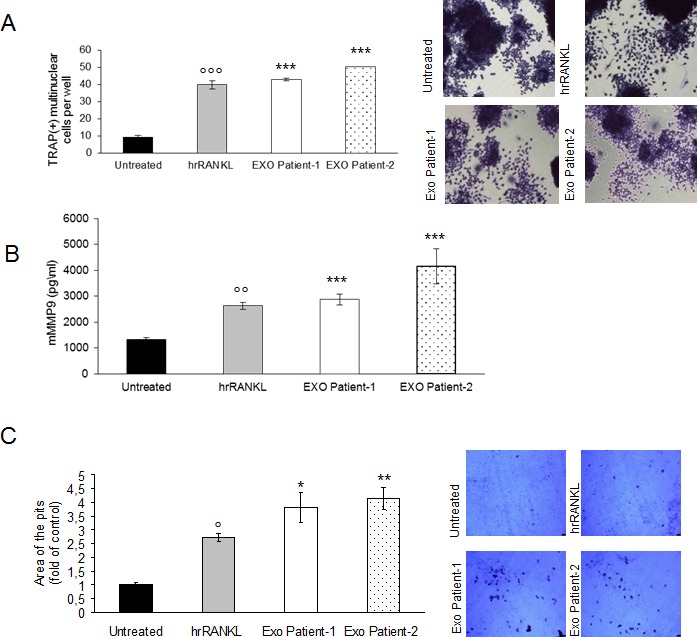
Exosomes in plasma of patients with multiple myeloma promote osteoclasts differentiation from Raw264.7 precursors and regulate bone resorption activity of osteoclasts **A.** Raw264.7 cells were incubated with 25ng\ml of hrRANKL (positive control) or with 25 μg/ml of exosomes isolated from plasma of two patients with MM for six days, and then stained for TRAP expression. TRAP positive cells (i.e., those containing three nuclei) were photographed and counted. Right panel shows representative pictures of Raw264.7 cells stained for TRAP (original magnification 20x). Data presented are the mean of three separate experiments. Sidak test:, EXO-Patient-1/-2 *vs* Untreated (^*^p < 0.05); °,hrRANKL *vs* Untreated (*p* < 0.05); **B.** Murine Metalloproteinase-9 (mMMP9) protein levels in the conditioned medium derived from Raw264.7 cells untreated or treated for six days with 25ng\ml of hrRANKL (positive control) or with 25 μg/ml of exosomes isolated from plasma of two patients with MM were determined by enzyme-linked immunoadsorbent assay (ELISA). Data presented are the mean of three separate experiments. Sidak test: ^*^,EXO-Patient-1/-2 *vs* Untreated (^*^p < 0.05); °,hrRANKL *vs* Untreated (°*p* < 0.05); **C.** Bone resorption ability of Raw264.7 cells treated for six days with 25ng\ml of hrRANKL (positive control) or with 25 μg/ml of exosomes isolated from plasma of two patients with MM, was evaluated by resorption pit assay on dentine discs. Data relative to the resorbed areas by mature osteoclasts are represented as fold increase of Raw264.7 cells untreated (black column, 1 arbitrary unit). Data presented are the mean of three separate experiments. Sidak test: ^*^,EXO-Patient-1/-2 *vs* Untreated (^*^p < 0.05); °,hrRANKL *vs* Untreated (*p* < 0.05); Right panel shows representative pictures of lacunae (dark areas) formed by cells treated with rhRANKL or exosomes from MM patients (original magnification 20x).

To further strengthen our results on the role of MM cell-derived exosomes on osteoclastogenesis, we assessed the effect of exosomes isolated from plasma of 2 MM patients on Raw264.7 cells differentiation. First, to characterize MM patient-derived exosomes, we performed Dynamic Light Scattering Analysis and we found that size of serum exosomes was in accordance with the reported size range of exosomes (Suppl. Figure 5C). We then examined the effect of MM patient-derived exosomes on osteoclastogenesis. Raw264.7 cells incubated with MM patient-derived exosomes for 6 days, differentiated into mature TRAP-positive multinucleated osteoclast (Figure 8A, left and right panel); in addition, secretion of mMMP9 increased significantly when Raw264.7 cells were exposed to exosomes (Figure 8B). We finally investigated if MM patient-derived exosomes played a role on the ability of mature osteoclasts to resorb bone. Notably, treatment with MM patient-derived exosomes significantly increased the formation of resorption pits as measured by the amounts of pits and overall area compared with untreated Raw264.7 cells (Figure 8C, left and right panel). Taken together, our results are in line with *in vitro* data using MM cell line-derived exosomes and demonstrate that MM patient-derived exosomes induce differentiation and modulate resorption activity of Raw264.7 cells.

### Multiple myeloma cell-derived exosomes affect osteoclastogenesis in human primary osteoclasts

**Figure 9:**
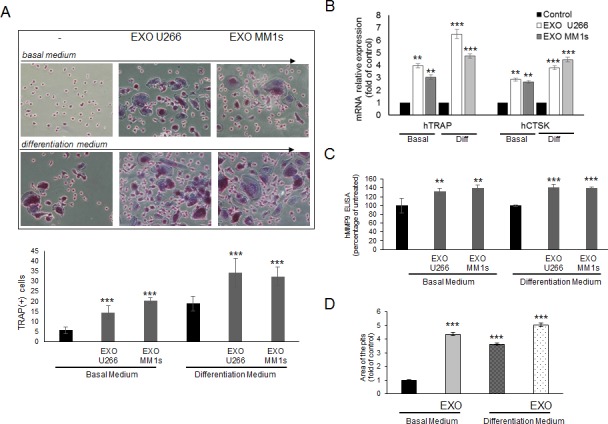
Exosomes produced by multiple myeloma cells induce osteoclast formation in human primary pOCs and promote formation of lacunae on dentine slices **A.** Human pOCs were cultured untreated or treated with 25 μg/ml of U266 or MM1s exosomes in basal medium and differentiation medium, and then stained for TRAP expression. TRAP positive cells were photographed and counted (lower panel). Upper panel shows representative pictures of human OCs stained for TRAP (original magnification 20x). Data presented are the mean of three different experiments. Sidak test: ^*^,EXO-U266/MM1s *vs* Untreated (^*^p < 0.05); **B.** Quantitative RT-PCR of hTRAP and hCSTK in human pOCs cultured untreated or treated with 25 μg/ml of U266 or MM1s exosomes in basal medium and differentiation medium. Values are expressed as fold of untreated cells (control) and are the mean of three different experiments. Sidak test: ^*^,EXO-U266/MM1s *vs* Untreated (^*^p < 0.05); **C.** ELISA assay of human MMP9 secreted in the conditioned medium derived from human OCs untreated or treated with 25 μg/ml of U266 or MM1s exosomes in basal medium and differentiation medium. Data presented are the mean of three separate experiments. Sidak test: *,EXO-U266/MM1s *vs* Untreated (*p < 0.05); **D.** Bone resorption ability of human OCs untreated or treated with 25 μg/ml of U266 or MM1s exosomes in basal medium and differentiation medium was evaluated by resorption pit assay on dentine discs. Data relative to the resorbed areas by mature OCs are represented as fold increase of human OCs untreated (black column, 1 arbitrary unit). Data presented are the mean of three separate experiments. Sidak test: *,EXO-U266/MM1s *vs* Untreated (*p < 0.05).

To evaluate the effects of MM cell-derived exosomes in a more physiological system, we analyzed the role of MM exosomes also on human primary osteoclasts. Both pOCs and mature OCs were prepared from human PBMCs obtained from three healthy different donors [26]; in detail, we studied the effects of U266- and MM1s- exosomes on differentiation of OCs cultured in complete OCs medium (differentiation medium) and in OCs medium lacking RANKL/MCS-F/dexamethasone (basal medium). As shown in Figure 9A, pOCs treated with U266- and MM1s- exosomes differentiated into large, multinucleated OCs, even in basal medium; the number of TRAP-positive mature OCs was significantly higher compared to control cells.

Accordingly, qRT-PCR analysis showed that treatment of human pOCs with U266 and MM1s exosomes increased the expression of TRAP and CSTK, as compared to control cells (Figure 9B); furthermore, ELISA assay revealed that human MMP9 protein secretion was increased in differentiated OCs treated with MM exosomes (Figure 9C). Furthermore, mature OCs treated with U266-exosomes strongly formed resorption pits on synthetic dentine discs, both in basal and differentiation medium (Figure 9D). We finally used as normal controls, exosomes derived from human peripheral blood mononuclear cells from healthy donors; osteoclastogenesis assays performed on both human pOCs and murine Raw264.7 cells showed that PBMCs-exosomes were not able to affect significantly OCs differentiation (Suppl. Figure 7).

## DISCUSSION

Multiple myeloma bone disease (MBD) is characterized by osteolytic bone destruction leading to impairment of patients's quality of life. Increased osteoclast activity and suppressed osteoblast function is the main mechanism of MBD. In detail, osteolytic lesions are consequential to osteoclasts recruitment, differentiation and function, which can be induced in the local bone marrow microenvironment by tumor cells; furthermore, osteoclastogenesis promote myeloma progression by both direct cell-cell communications as well as indirectly by soluble factors released by local bone marrow microenvironment cells [27]. Within the tumor microenvironment, the release of exosomes from cancer cells may confer relevance in cancer growth and progression [2, 21].

In the current study, we focused on the role played by MM cell-derived exosomes, as well as by MM patient's exosomes, on the process of bone remodelling by performing *in vitro* assay with both human primary osteoclasts and murine pre-osteoclastic Raw264.7 cells. Exosomes from three different MM cell types (U266, MM1s and OPM2) and from MM patients were isolated and characterized for their biochemical and dimensional properties and the results obtained were in agreement with those from other groups [24, 28]. We also showed that MM exosomes were able to be actively internalized by Raw264.7 cells, while uptake inhibition in the presence of 5-ethyl-N-isopropyl amiloride (EIPA) suggested the involvement of additional endocytic pathways [29, 30].

Recent data reported that human multiple myeloma cells secrete microvesicles able to promote *in vitro* and *in vivo* angiogenesis by stimulating the endothelial cells to proliferate, invade and secrete IL-6 and VEGF [31]. In the bone marrow niche, OCs develop and function in close association with microvascular endothelial cells which, in turn, are critically involved in bone development and remodelling, influencing OCs recruitment, formation and activity [32]. The crosstalk between bone marrow stromal cells (BMSCs) and MM cells supports the proliferation, survival, migration, and drug resistance of MM cells as well as osteoclastogenesis and angiogenesis [34,35]. Several factors produced as a result of MM-BMSC cell interactions alter OCs and OBs activities; MM cells directly stimulate OCs differentiation by secreting OC-activating factors and, indirectly, by stimulating BMSCs secretion of additional OC-activating factors [33-35]. Roccaro AM and colleagues have recently demonstrated that BMSCs of patients with MM release exosomes that may be transferred to MM cells thus inducing MM cell growth and promoting dissemination. The proteomic analysis of MM BMSCs exosomes evidenced elevated expression of oncogenic proteins, cytokines and protein kinases compared with exosomes from normal BMSCs [36].

In another study, exosomes obtained from BMSCs induced survival and drug resistance of human MM cells and the authors speculated on the possibility that BMCSs and myeloma cells may also communicate through mutual exchange of exosomes [22]. Recently, it has been showed that multiple myeloma cells in chronic hypoxic bone marrow secrete more exosomes than the parental cells under normoxia; interestingly, exosomal miR-135b regulated the HIF-1 signaling in HUVECs by enhancing angiogenesis [37]. These findings acquire greater importance if we consider that hypoxia triggers angiogenic events within the microenvironment [38] and that inhibition of hypoxia in MM cells impair pro-osteoclastogenic cytokines expression with a significative reduction of bone loss [39]. Furthermore, the analysis of deregulated microRNAs, as well as of non-coding RNAs [40] is emerging as a novel approach to disclose the regulation of tumor suppressor or tumor promoting pathways in tumor cells [41-44].

Overall, our results here summarized support the hypothesis that exosomes are important mediators of crosstalk between MM cells and BM microenvironment. The findings described in our study, provide the first evidence, at our knowledge, that exosomes derived from myeloma cells directly influence OCs differentiation and function. Bone resorption is the result of sequential stages characterized by pOCs migration, invasion and homing from the peripheral circulation to bone, where eventually takes place differentiation of pre-OCs into multinucleated OCs, responsible of bone remodelling. Notably, in MBD, myeloma cells exert a significant effect on recruitment, proliferation and differentiation of OC progenitors [26, 45]. Even if the mechanism of osteoclast precursor recruitment remains poorly understood, several chemoattractants have been show to play critical role in controlling the migration of monocyte-lineage precursors from peripheral circulation to sites of bone resorption [46]. Consistent with these observations, we investigated the potential role of MM cell-derived exosomes on migration of pOCs cells. Our findings clearly indicated that exosomes isolated by U266 and MM1s cells influenced positively both murine and human pOCs migration, inducing a strong expression of CXCR4 in human pOCs. OPM2-cell derived exosomes exerted minor effects on the phenomena described above. In addition, OPM2-cell derived exosomes affected osteoclast differentiation and function more weakly compared to U266/MM1s exosomes. This could be explained by well-known heterogeneity in myeloma [47]. Since MM cells can colonize bone marrow microenvironment where they induce osteolytic lesions, we investigated the potential role of MM cell-derived exosomes on OCs differentiation. To address this issue, we analyzed the expression of osteoclast-specific markers in Raw264.7 cells cultured for 6 days with MM cell-derived exosomes or with RANKL, a well-known inducer of OCs differentiation, used as positive control [13]. In parallel, we evaluated the effects of MM cell-derived exosomes on human primary pOCs cultured in basal or complete OCs medium. In particular, we examined the expression of CTSK, a proteolytic enzyme responsible of bone resorption, MMP9, a gelatinase secreted by OCs mature involved in bone resorption, and, finally, TRAP, a regulator of bone resorption through degradation of bone matrix phosphoproteins, highly expressed in terminally differentiated OCs [48-50].

We found that the expression of OCs markers, such as secretion of mMMP9, was significantly increased in Raw264.7 cells and human OCs exposed to MM cell-derived exosomes; these results demonstrate that in addition to affect migration of osteoclast precursors, MM cell-derived exosomes induce their differentiation in mature OCs and control secretion of factors involved in bone resorption activity. Data from literature reported that MM cells stimulate OCs development from precursor cells as well as OCs activity through production of key osteoclastogenic factors [27, 51]. However, a direct effect of MM cells on recruitment and differentiation of pOCs has not clearly demonstrated. In our study, addition of multiple myeloma derived exosomes to osteoclast precursors resulted in formation of functional osteoclasts. These latter were evidenced by the presence of multinucleated TRAP-positive cells and characterized by OCs actin rings, indicative of unique cell adhesion structures established at sites of OCs attachment to the bone surface [52-54]. Notably, OCs, formed following treatment with MM-derived exosomes, resemble mature resorptive cells able to excavate lacunae *in vitro*, similar to the structures formed when the cells degrade bone *in vivo* [55]. Of importance, the results obtained appear to be cell-type specific, as exosomes derived by the metastatic colorectal cancer cell line SW620 did not elicit the same effects.

Several studies showed that pathways correlated with OCs differentiation promote OCs survival, activate PI3-Kinase/AKT signaling pathway and increase the expression of anti-apoptotic genes [56, 57]. Interestingly, Szymczyk KH et al demonstrated that active caspase-3 is required for OCs differentiation, suggesting a non-apoptotic role for caspase-3 in pOC cells. In particular, authors showed that caspase-3 is an early signalling event that affect TRAP expression and they observed a decreased number of functional OCs in procaspase-3 knockout mice as well as the lack of differentiation in OCs in response to RANKL when caspase-3 activity was inhibited [25]. In our study, we provided evidence that MM cell-derived exosomes induced caspase-3 activation in Raw264.7 cells, even in absence of a clear apoptosis; notably, treatment with MM cell-derived exosomes fails to induce TRAP activity in Raw264.7 cells that were pharmacologically inhibited for caspase-3 activity.

Furthermore, MM derived exosomes activated AKT pathway which in turn, as it is well known, enhanced survival and anti-apoptotic gene expression in OCs. Importantly, our *in vitro* observations were confirmed *ex vivo* by using exosomes isolated from MM patients. Exosomes isolated and characterized from plasma of MM patients in fact positively modulated the differentiation and activity of OCs, thus adding a potential clinical significance to our results.

An important question that remains to be addressed is to understand how MM cell-derived exosomes may act on osteoclastogenesis; to address this issue, future studies will be necessary to identify the exosomal factors responsible for the processes we described.

Notably, a recent study described the proteomic content of exosomes derived from MM1s and U266 cell lines, comparing the protein identified between the vesicles and cellular lysates of each cell line [58]. The authors detected the exclusive presence in the exosomal compartment of bone marrow stromal protein 2 (BST-2), previously described as strongly expressed in bone metastatic breast cancer [59]. Taken together the results reported in this study significantly enhance our understanding about intercellular communication in MBD by demonstrating that MM cells release biologically active exosomes responsible, inside the metastatic niche, of pOCs recruitment, migration and differentiation also through apoptotic inhibition.

## MATERIALS AND METHODS

### Cell lines and reagents

U266, MM1s and OPM2 cell lines were purchased from ATCC^®^ (LGC Standards S.r.l.Sesto San Giovanni, MI, Italy) and grown in RPMI-1640 (Gibco, Life Technologies, USA) supplemented with 10% Fetal Bovine Serum (FBS, Lonza Group, Basel, Switzerland).

Murine macrophage Raw264.7 cells were purchased from ATCC^®^ and cultured in Dulbecco's modified Eagle's medium (DMEM), supplemented with 10% FBS. To induce differentiation, cells were treated with 25ng/ml of human recombinant RANK Ligand (Gibco, Life Technologies, USA) for 6 days in DMEM, supplemented with 10% FBS, previously ultracentrifugated (Raw264.7 cells OC medium). Alternately, cells were treated for 6 days with 25μg/ml of MM cell-derived exosomes, in DMEM, supplemented with 10% of ultracentrifugated FBS (see below). 5-(N-Ethyl-N-isopropyl) amiloride (EIPA) was purchased from Sigma Aldrich-Italy. Caspase-3 inhibitor Z-DEVD-FMK was purchased from R&D systems.

### Isolation of human peripheral blood mononuclear cells

Human blood samples were obtained from healthy donors, after written informed consent obtained in accordance with the Declaration of Helsinki guidelines and University of Palermo Ethics committee. Human peripheral blood mononuclear cells (PBMCs) were isolated using the Ficoll-Paque (GE Healthcare Bio Science, Uppsala, Sweden) separation technique.

### Preparation of human primary pOC and OCs

PBMCs were cultured at 2.5×10^6^cells/ml α-MEM supplemented with 10% FBS previously ultracentrifuged, 25ng/ml of human recombinant RANK Ligand (Gibco, Life Technologies, USA), 25ng/ml of human M-CSF (Gibco, Life Technologies, USA), and 10nM dexamethasone (Sigma-Aldrich Italy) (Human OC medium). After 2-4 days, the culture were washed with α-MEM medium to remove non adherent cells. The remaining cells were mononucleated, expressed TRAP and were considered committed pre-osteoclast cells. For human osteoclastogenesis assays, OC medium was added and the cultures were continued for additional 6-10 days, at the end of the period they contained large mature multinucleated OCs. The culture period was 16 days for both TRAP staining assay, bone resorption assay and qRT-PCR analysis.

### Exosome purification

Exosomes released by both multiple myeloma cells (U266, MM1s and OPM2 cells) during a 48 h culture period and PBMCs during a 24h culture period, were isolated from conditioned culture medium supplemented with 10% FBS previously ultracentrifuged by differential centrifugations as previously described [60]. To confirm vesicles identity as exosomes, vesicles were purified on a 30% sucrose/D2O cushion. Vesicles contained in the sucrose cushion were recovered, washed, ultracentrifuged for 90 min in PBS and collected for use. Exosome protein content was determined by the Bradford assay [61]. Exosomes isolated from human blood samples were obtained from two diagnosed MM patients. Informed consent was obtained from patients, according to the Declaration of Helsinki and with hospital Ethics Committee approval. Serum were isolated using the Ficoll-Paque (GE Helthcare-Bio Science, Uppsala, Sweden) separation technique, according to the manufacturer's instructions. Exosomes isolated from human serum were prepared as described above. The activity of acetylcholinesterase, an exosome marker protein, was determined as described by Savina et al. [24].

### Uptake of multiple myeloma exosomes by Raw264.7 cells

MM cell-derived exosomes (isolated from U266, MM1s and OPM2) were labeled with PKH26 (Sigma-Aldrich, Italy), according to the manufacturer's instructions. Briefly, exosomes collected after the 100,000×g ultracentrifugation, were incubated with PKH26 for 10 min at room temperature. Labeled exosomes were washed in PBS, centrifugated, resuspended in low serum medium and incubated with Raw264.7 cells for 1-4 hours at 37°C or 4°C. In a set of experiments, Raw264.7 cells were pretreated with 25 μM EIPA, a known inhibitor of exosomes uptake, for 1 hour. After incubation, cells were processed as previously described [60]. Raw264.7 cells were stained with ActinGreen^TM^ 488 Ready Probes^R^ Reagent (Life Technologies, USA) that binds F-actin with high affinity. Nuclei were stained with Hoechst (Molecular Probes, Life Technologies, USA) and analysed by confocal microscopy (Nikon Eclipse T*i*).

### Western blotting and antibodies

SDS-PAGE Electrophoresis and Western Blotting were performed as previously described [62]. Briefly, cells were lysed for an 1 hr in lysis buffer containing 15mM Tris/HCl pH7.5, 120mM NaCl, 25mM KCl, 1mM EDTA, 0.5% Triton X100, and Protease Inhibitor Cocktail (100X, Sigma–Aldrich, USA). Cell lysates (30μg per lane) were separated using 4-12% Novex Bis-Tris SDS-acrylamide gels (Invitrogen, Life Technologies, USA), transferred on Nitrocellulose membranes (Invitrogen, Life Technologies, USA), and immunoblotted with the primary antibodies. The antibodies against the following proteins were used: Bcl-XL (sc-8392), survivin (sc-17779), MMP9 (sc-6840), CD63 (sc-15363), Calnexin (sc-23954), β-actin (sc-47778) and all secondary antibodies were obtained from Santa Cruz Biotechnology (Santa Cruz Biotechnology, Inc., Santa Cruz, CA, USA); Phospho-Akt (Ser473), Akt (pan) (C67E7), Alix (2171S) were obtained from Cell Signaling (Beverly, MA); Cathepsin K (ab 19027) was obtained from Abcam (Abcam, Cambridge, UK). Chemiluminescence was detected using Amersham^TM^ ECL^TM^ Western Blotting Detection Reagents (GE Healhtcare, UK).

### Dynamic Light Scattering (DLS) analysis

Exosome size distribution was determined by DLS experiments. Collected exosome samples were diluted 30 times to avoid inter-particle interaction and placed at 20°C in a thermostated cell compartment of a Brookhaven Instruments BI200-SM goniometer, equipped with a solid-state laser tuned at 532 nm. Scattered intensity autocorrelation functions g2(t) were measured by using a Brookhaven BI-9000 correlator and analyzed in order to determine the distribution P(D) of the diffusion coefficient D by using a constrained regularization method or alternatively a gamma distribution [28]. The size distribution, namely the distribution of hydrodynamic diameter Dh, was derived by using the Stokes-Einstein relation: D = (kBT)/(3πη Dh), where D is the diffusion coefficient, kB is the Boltzman constant, η is the medium viscosity and T is the temperature. The mean hydrodynamic diameter of exosomes was calculated by fitting a Gaussian function to the measured size distribution.

### Cell viability

Cell viability of Raw264.7 cells stimulated with MM cell-derived exosomes was evaluated by MTT (Sigma Aldrich, Italy). Raw264.7 cells were seeded in 96-well plates at a density of 5000 cells/cm^2^ per well and then cultured with MM cell-derived exosomes, hrRANKL or a MM-conditioned medium that was previously deprived of exosomes. Viability at different time points (72 hours and 7 days) was assessed by measuring the absorbance at 450 nm using an ELISA plate reader (EL800 Absorbance Microplate Reader, BioTek Instruments, U.S).

### Migration assay

Migration was determined using 8μ-pore filters for the Transwell migration assay (BD Biosciences), according to the manufacturer's instructions and as previously described [63]. Briefly, murine Raw264.7 cells and human pre-osteoclast cells (1×10^5^ cells/well) treated for 24hrs with MM cell-derived exosomes (25μg/ml) were washed and resuspended in DMEM high glucose medium supplemented with 1% of ultracentifuged FBS. Cells were then placed in the upper migration chambers, whereas the lower chamber contained DMEM high glucose medium supplemented with 10% ultracentifugated FBS. Filters were removed after 24 hours, fixed in methanol and cells stained with Diff-Quick (Medion Diagnostics GmbH, Düdingen, Switzerland). Alternatively, pre-osteoclast cells were not treated and MM cell-derived exosomes (25μg/ml) were added directly in the lower chamber contained DMEM high glucose medium supplemented with 10% ultracentifugated FBS. Two independent experiments were performed in triplicate; cells from 5 different fields were counted for each condition.

### RNA extraction and real-time PCR

Total RNA from both Raw264.7 cells and Human primary osteoclast cells was extracted using TRIzol Reagent, (Invitrogen, Life Technologies, USA) according to the manufacturer's protocol. qRT–PCR was used to confirm the expression levels of mRNAs. For mRNA detection, oligo-dT-primed cDNA was obtained using the High-Capacity cDNA Reverse Transcription Kits (AB Applied Biosystems, USA) and then used as template to quantify mRNA levels by Fast SYBR^R^ Green Master Mix (AB Applied Biosystem, USA). Primers are reported in Supplemental Table 1. Relative changes in gene expression between control and treated samples were determined with the ΔΔCt method. Final values were expressed as fold of induction.

### TRAP staining assay

Human primary osteoclasts and Raw264.7 cells cultured in OC medium alone or with MM cell-derived exosomes (25μg/ml) were stained for detection of tartrate-resistant acid phosphatase (TRAP) activity, according to the manufacturer's protocol (Acid Phosphatase, Leukocyte (TRAP) Kit; Sigma–Aldrich, USA) and evaluated by light microscopy. Multinucleated TRAP+ cells containing more than three nuclei were scored as mature osteoclasts. Three independent experiments were performed in triplicate; cells from 5 different fields were counted for each condition.

### Bone resorption assay

Human primary osteoclasts and Raw264.7 cells were seeded at a density of 5×10^4^ cells/ml in 96-well plates on organic dentine discs (Dentine Discs; Pantec, Torino, Italy) and cultured in OC medium alone or with MM cell-derived exosomes (25μg/ml). The dentine discs, at the end of the culture duration, were rinsed with 70% sodium hypochlorite and fixed in 4% glutaraldehyde. The resorption pits were stained using 1% toluidine blue and observed with a light microscope (Leica DM2500 Microsystems, Germany) at a 10X magnification. Three fields of each dentin disc for each experimental point were scored in three independent experiments. The number of the pits was calculated by NIH imageJ software analysis (htttp:/rsbweb.nih.gov/ij/). Surface of dentin samples have been observed using Zeiss EVO DH15 scansion electron microscopy (SEM).

### Cytoskeletal organization

To assess cytoskeleton organization, Raw 264.7 cells were seeded on coverslips, cultured for 6 days with either hrRANKL (25ng/ml) or MM cell-derived exosomes (25μg/ml), fixed in 4% paraformaldehyde and stained with phalloidin–Alexa fluor 488 (Life Technologies, USA). Analysis was performed at confocal microscopy (Nikon Eclipse T*i*).

### Caspases assay

Human primary osteoclasts and Raw264.7 cells cultured for 24h in OC medium alone or with 25μg/ml MM cell-derived exosomes were analyzed for activity of caspase 3/7 by Caspase-Glo^®^ 3/7 Assay System (Promega, Mannheim, Germany) according to manufacturer's instructions. Fluorescence and luminescence was recorded using GloMax Multi+microplate reader (Promega, Mannheim, Germany). Caspase-3 activity was pharmacologically inhibited using 50μM Z-DEVD-FMK (R&D systems). The inhibitor was added 1h before and during osteoclast differentiation treatment. These inhibitors are peptide substrates that compete for active site binding based on the substrate preference of caspase-3.

### Annexin V apoptosis assays

Annexin V apoptosis assays were performed using Annexin V-FITC conjugated (Life Technologies, USA). The stained cells were immediately analyzed on a CyFlow Space cytometer (Partec CyFlow^R^ Space Flow cytometer).

### Flow cytometric analysis

Human primary osteoclasts treated for 24h with MM cell-derived exosomes (25μg/ml), were stained with PE-conjugated mouse anti-Human CXCR4 (clone 12G5, R&D System). The stained cells were immediately analyzed on a CyFlow space cytometer (Partec CyFlow^R^ Space Flow cytometer).

### ELISA assay

MMP9 levels secreted by both Human primary OCs and Raw264.7 cells were quantified respectively by Human MMP-9 ELISA assays (Invitrogen) and mouse TOTAL MMP9–enzyme-linked immunosorbent assays (R&D Systems, Minneapolis, MN).

### Statistical analysis

The statistical analysis was performed using the IBM SPSS Statistics 21 software. Data are reported as mean ± standard deviation (SD) at a significant level of p < 0.05. After having verified normal distribution (Kolmogroc-Smirnov test) and homoscedasticity (Levene test) of data, the influence of MM cell-derived exosome on various parameters of murine macrophage Raw264.7 cells was investigated by a generalized linear model (GLM) for repeated measures with ‘experiment replication’ (n=3) as a within-subject factor and ‘groups’ as between-subject factor, followed by adjusted Sidak multiple comparison test.

## SUPPLEMENTARY MATERIALS, FIGURES, TABLES


