# Supplementary material for: Involvement of multiple myeloma cell-derived exosomes in osteoclast differentiation
Source: Oncotarget. 2015 Apr 12;6(15):13772–89. doi: 10.18632/oncotarget.3830 (PMC4537049; doi:10.18632/oncotarget.3830)
Supplement: Supplementary file 1 [file oncotarget-06-13772-s001.pdf]

Involvement of multiple myeloma cell-derived exosomes in osteoclast differentiation

Supplementary Material

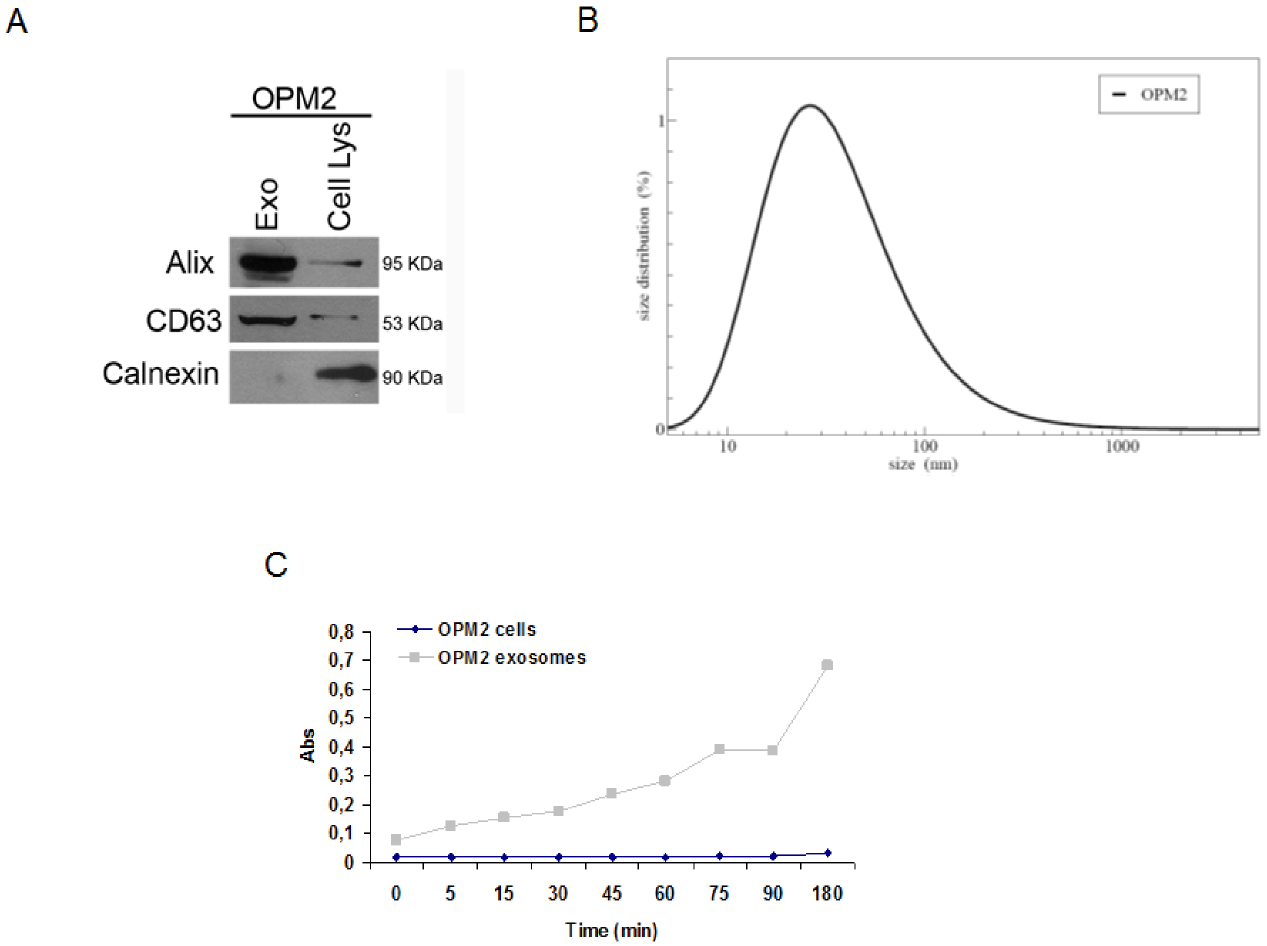

Supplemental Figure 1: **Characterization of exosomes released by OPM2 cells** (**a**) Western blotting analysis of Alix, CD63 and Calnexin in OPM2-derived exosomes and cellular lysates. (**b**) Dynamic light scattering (DLS) analysis of OPM2-derived exosomes (**c**) Acetylcholinesterase assay of exosomes and total cell lysated obtained from OPM2 cells.

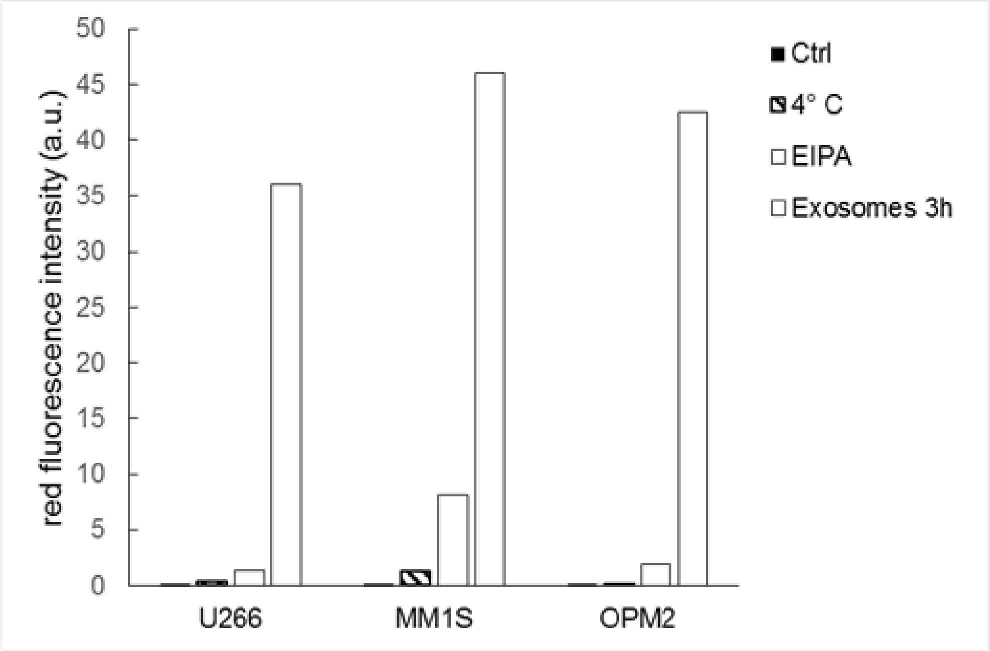

Supplemental Figure 2: Semi-quantitative analysis of up-take assay (**a**) Semi-quantitative analysis of PKH-26 (Red) fluorescence intensity in the cytoplasm of RAW264.7 cells treated with 25 µg/ml of exosomes released by U266, MM1S and OPM2 compared with control cells incubated at 37° C or 4°C, for 3 hours. White bar shows Raw264.7 cells treated with 25 µg/ml of exosomes and 25 µM of EIPA, for 3 hours.

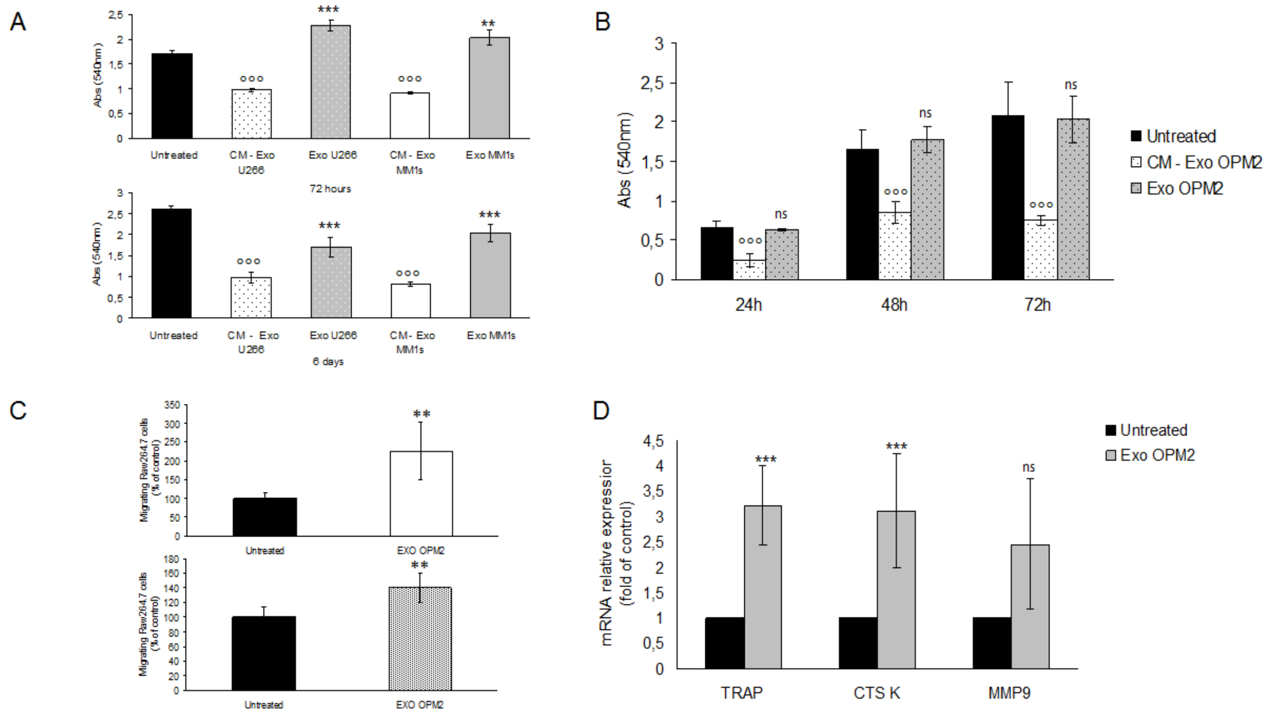

Supplemental Figure 3: Effect of MM cell-derived exosomes on osteoclast precursors viability, migration and expression of osteoclast specific markers a) Raw264.7 cells were cultured for 72 hours (upper panel) or six days (lower panel) treated or not with 25 µg/ml of U266 and MM1s exosomes or incubated with a MM conditioned medium exosomes-deprived (CM- Exo U266/MM1s). Cell viability was assessed by MTT assay. Averaged values of three independent experiments are plotted including ±S.D. Sidak test: *,EXO-U266/MM1s vs Untreated ($^{*}p < 0.01$); CM-EXO U266/MM1s vs Untreated (°p < 0.05); (**b**) RAW 264.7 cells were cultured for 24, 48 and 72 hours untreated or treated with 25 µg/ml of OPM2 exosomes or incubated with a MM conditioned medium exosomes-deprived (CM- Exo OPM2). Cell viability was assessed by MTT assay. Averaged values of three independent experiments are plotted including ±S.D. Sidak test: *,EXO-OPM2 vs Untreated ($^{*}p < 0.05$); °,CM-OPM2 vs Untreated (°p < 0.05); (**c**) Migration assay of Raw264.7 cells untreated or pretreated for 24 hours with 25 µg/ml of OPM2 exosomes (upper panel). In the lower panel is shown the motility of Raw264.7 cells stimulated by addition of 25 µg/ml of OPM2 exosomes in the bottom chamber of the transwell. Sidak test: *,EXO-OPM2 vs Untreated ($^{*}p < 0.01$); (**d**) Quantitative RT-PCR of mTRAP, mCTSK and mMMP9 in Raw264.7 cells untreated or treated for six days with 25 µg/ml of OPM2 exosomes. Raw Ct were normalized to GAPDH housekeeping gene and expressed as fold increase of untreated (black column, 1 arbitrary unit). Columns, means; Bars, S.D. Values represent mean of three different experiments. Sidak test: *,EXO-OPM2 vs Untreated ($^{*}p < 0.05$).

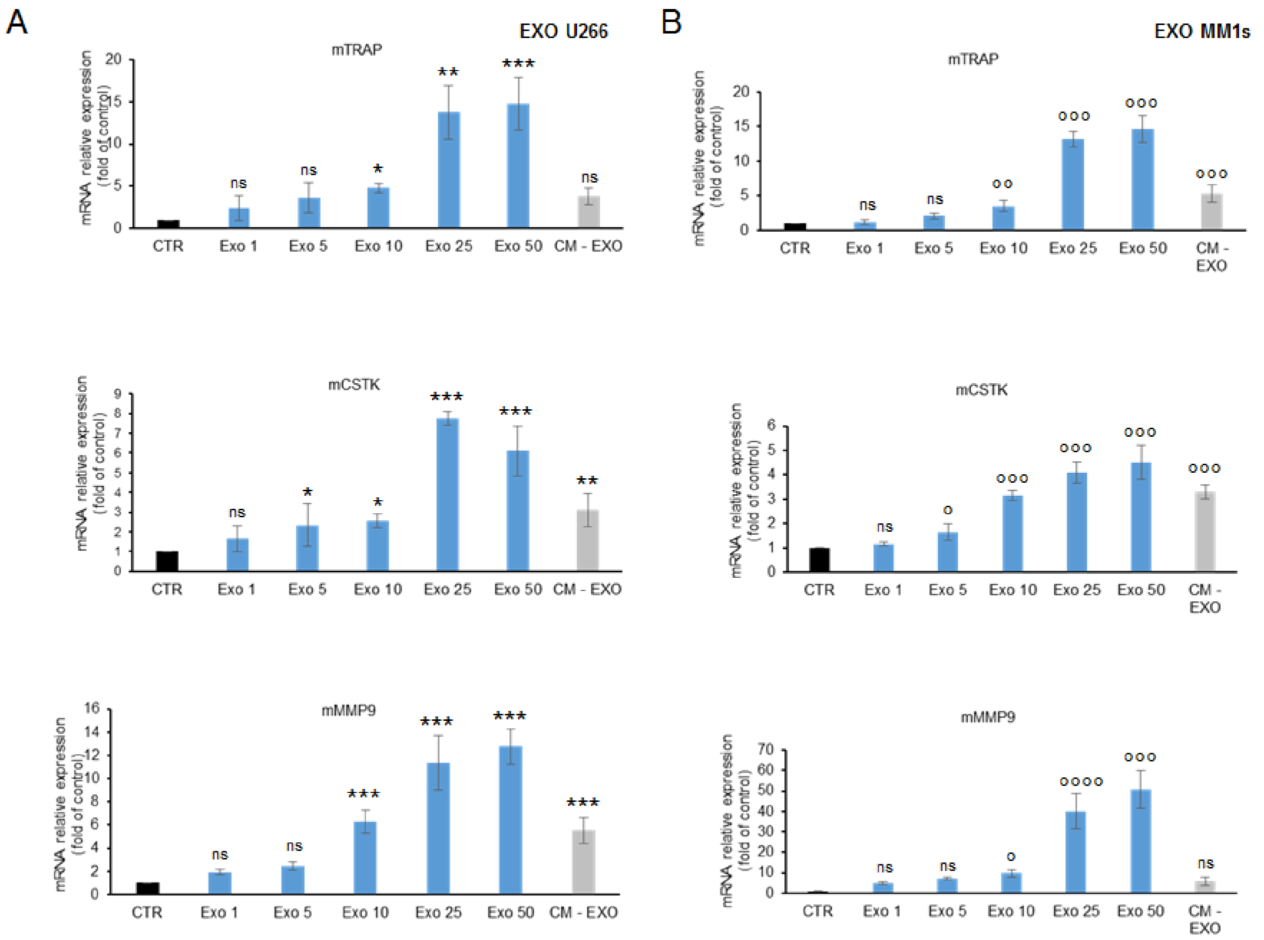

Supplemental Figure 4: **Dose-dependent effect of U266- and MM1s-derived exosomes on OCs specific markers expression** (**a**) Quantitative RT-PCR of mTRAP, mCTSK and mMMP9 in Raw264.7 cells untreated, treated with a conditioned medium exosome deprived and with different doses of U266 exosomes. Values are expressed as fold of control and are the mean of three different experiments. Sidak test: *,EXO-U266 vs Untreated ($^{*}p < 0.05$); **;** (**b**) Quantitative RT-PCR of mTRAP, mCTSK and mMMP9 in Raw264.7 cells untreated, treated with a conditioned medium exosome deprived and with different doses of MM1s exosomes. Values are expressed as fold of control and are the mean of three different experiments. Sidak test: $^{\circ}$,EXO-MM1s vs Untreated ($^{\circ}p < 0.05$).

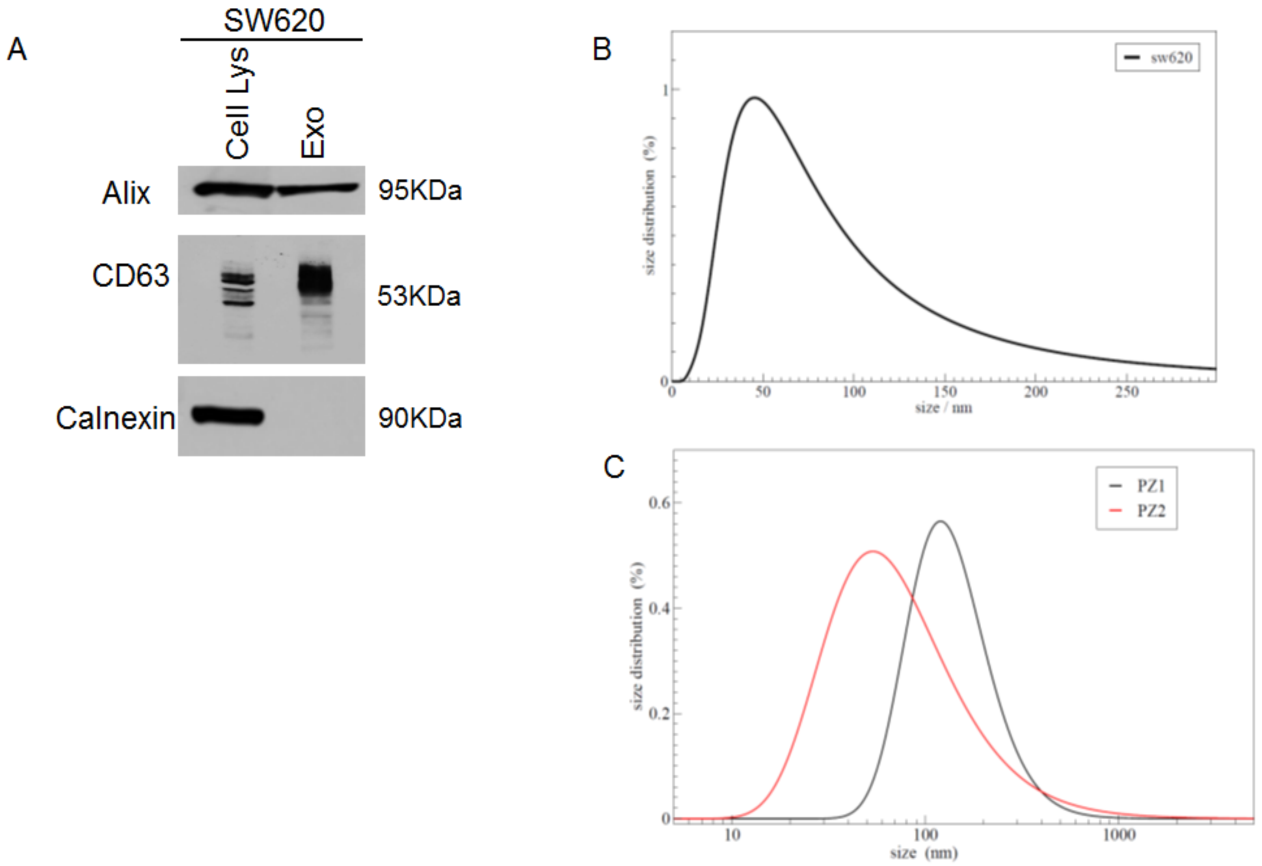

Supplemental Figure 5: **Characterization of exosomes from colorectal SW620 cell line and from plasma of patients with multiple myeloma** (**a**) Western blotting analysis of Alix, CD63 and Calnexin in both SW620-derived exosomes and cellular lysates. (**b**) Dynamic light scattering (DLS) analysis of SW620-derived exosomes (**c**) Dynamic light scattering (DLS) analysis of exosomes isolated from plasma of two patients with MM (Patient-1/2). Results were plotted as a % mass distribution in order to accurately represent the size distribution of the biological sample.

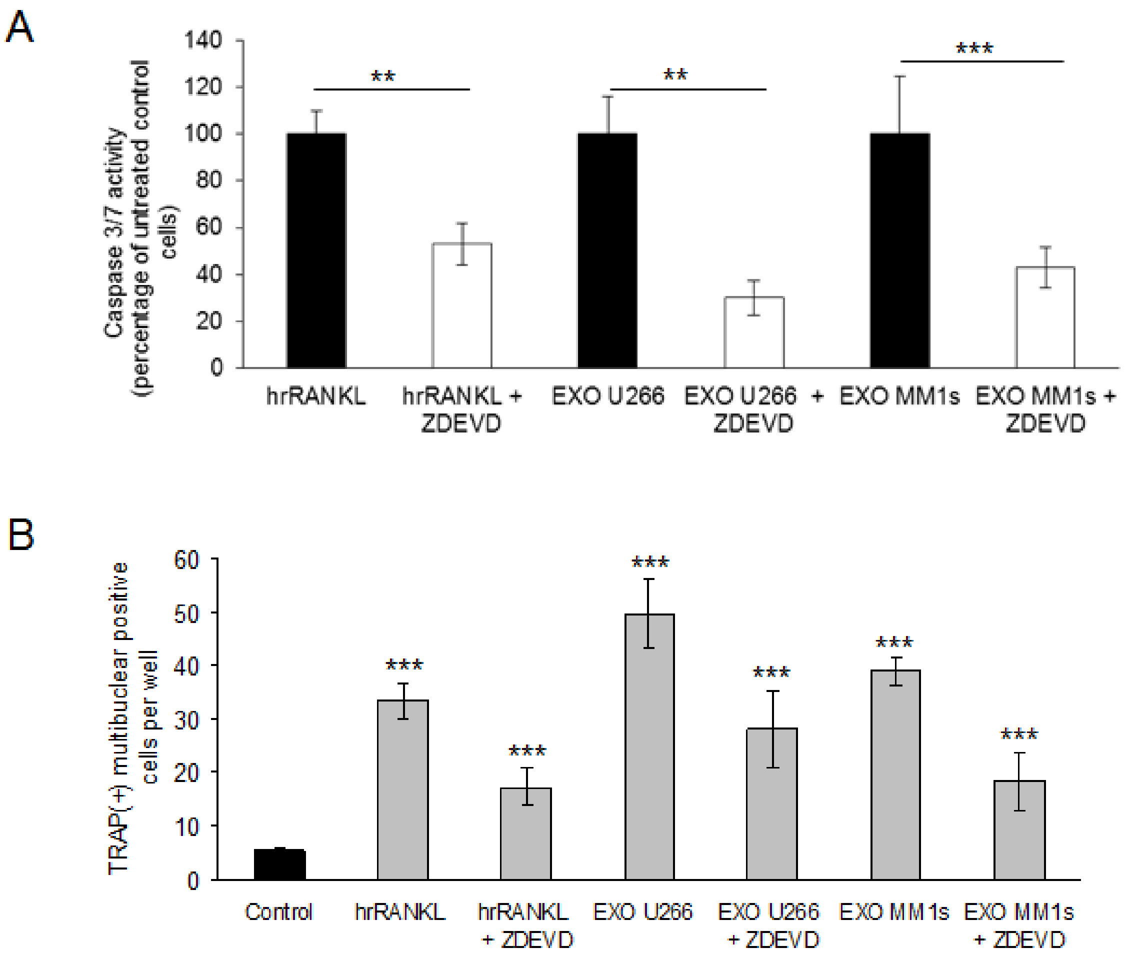

Supplemental Figure 6: **Inhibition of caspase-3 activity decreases Raw264.7 cell differentiation in response to MM cell-derived exosomes** (a) Caspase 3/7 activity assay was analyzed in Raw264.7 cells untreated or pretreated with a caspase-3 inhibitor (50µM Z-DEVD-FMK) 1h before and during hrRANKL or MM-exosomes treatment. Results are expressed as percentage of caspase activity compared to untreated cells (b) Raw264.7 cells untreated or pretreated with a caspase-3 inhibitor (50µM Z-DEVD-FMK) 1h before and during hrRANKL or MM-exosomes treatment were stained for TRAP expression. TRAP positive cells were photographed and counted. Data presented are the mean of three different experiments. Sidak test: *,EXO-U266/MM1s + ZDEVD vs EXO-U266/MM1s (*$p < 0.05$); *,hrRANKL+ZDEVD vs hrRANKL (*$p < 0.05$).

A

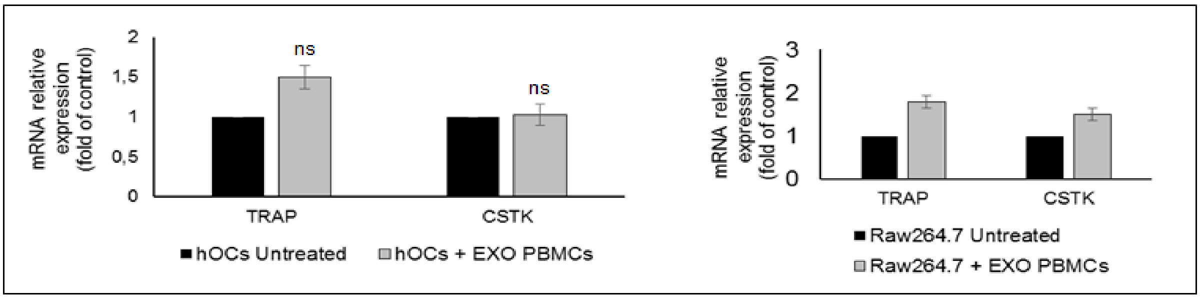

B

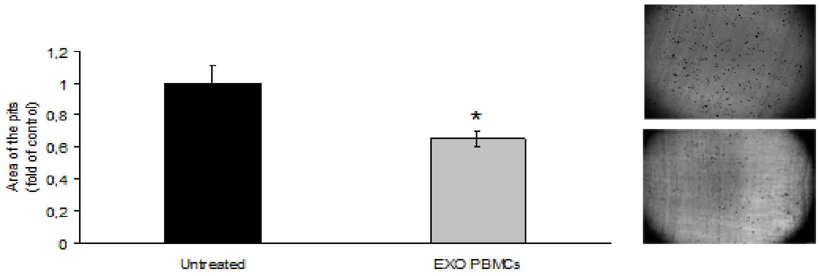

Supplemental Figure 7: **Effect of PBMCs derived exosomes on osteoclast differentiation** (**a**) Quantitative RT-PCR of TRAP and CSTK in human pOCs (left panel) and Raw264.7 cells (right panel) cultured untreated or treated with 25 µg/ml of PBMCs exosomes. Values are expressed as fold of control and are the mean of three different experiments. Sidak test: *,EXO-PBMCs vs Untreated (*$p < 0.05$); (**b**) Bone resorption ability of human OCs untreated or treated with 25 µg/ml of PBMCs exosomes in differentiation medium was evaluated by resorption pit assay on dentine discs. Data relative to the resorbed areas by mature OCs are represented as fold increase of human OCs untreated. Data presented are the mean of three separate experiments. Sidak test: *,EXO-PBMCs vs Untreated (*$p < 0.05$).

Supplemental Table 1: Semi-quantitative analysis of PKH-26 (Red) fluorescence intensity in the cytoplasm of RAW264.7 cells treated with 25 µg/ml of exosomes released by U266, MM1S and OPM2 compared with control cells incubated at 37° C or 4°C, for 3 hours. White bar shows Raw264.7 cells treated with 25 µg/ml of exosomes and 25 µM of EIPA, for 3 hours.

Gene	Forward primer sequence (5'-3')	Reverse primer sequence (5'-3')
mouse GAPDH	CCCAGAAGACTGTGGATGG	CAGATTGGGGGTAGGAACAC
mouse TRAP	GCGACCATTGTTAGCCACATACG	CGTTGATGTCGCACAGAGGGAT
mouse CathK	GCGTTGTTCTTATTCCGAGC	CAGCAGAGGTGTGTACTATG
mouse MMP9	GCTGACTACGATAAGGACGGCA	GCGGCCCTCAAAGATGAACGG
human β-actin	AGGCACCAGGGCGTGAT	GCCCACATAGGAATCCTTCTGAC
human TRAP	GATCCTGGGTGCAGACTTCA	GCGCTTGGAGATCTTAGAGT
human CathK	ACCGGGGTATTGACTCTGAA	GAGGTCAGGCTTGCATCAAT
human MMP9	CGCTACCACCTCGAACTTTG	GCCATTCACGTCGTCCTTAT
